# Establishment of a meta-analysis based novel aortic dissection mouse model

**DOI:** 10.1038/s41598-022-25369-x

**Published:** 2022-12-12

**Authors:** Hongcheng Jiang, Wanjun Liu, Xingwei He, Hesong Zeng

**Affiliations:** 1 Division of Cardiology, Department of Internal Medicine, Tongji Hospital, Tongji Medical College, Huazhong University of Science and Technology, Wuhan, China; 2 Hubei Provincial Engineering Research Center of Vascular Interventional Therapy, Wuhan, China

**Keywords:** Cardiology, Diseases

## Abstract

Aortic dissection (AD) is a life-threatening disease and the detailed mechanism remains unclear. Thus, proper animal models are urgently required to better understand its pathogenesis. Our current study aims to establish a reliable, time and cost-effective mouse AD model. To conduct the meta-analysis, we searched PubMed for related studies up to 2021 and statistical analysis was conducted using Review Manager 5.4. For the animal experiment, 6-week-old male ApoE^−/−^ mice were given β-aminopropionitrile (BAPN) at a concentration of 1 g/L for 3 weeks before being infused with saline, 1000 ng/kg/min or 2500 ng/kg/min angiotensin II (AngII) via osmotic mini pumps for 2 or 4 weeks. To determine the presence of AD, we performed B-ultrasonography, hematoxylin and eosin (H&E) staining, and van Gieson staining. The result of the meta-analysis showed that the use of BAPN and more than 2000 ng/kg/min AngII can increase the rate of AD formation, whereas administrating Ang II for more than 28 days has no significant effect on the rate of AD formation when compared with the less than 14 days group. In the present study, mice treated with BAPN combined with 2500 ng/kg/min AngII for 2 weeks (12/20) had a significantly higher AD formation rate than mice treated with BAPN combined with 1000 ng/kg/min Ang II for 4 weeks (2/10), and had a similar model formation rate compared with the mice treated withβ-aminopropionitrile combined with 2500 ng/kg/min AngII for 4 weeks (6/10). There were 3 mice (3/10) and 6 mice (6/20) who died in the group treated with β-aminopropionitrile combined with 2500 ng/kg/min AngII for 4 weeks and 2 weeks respectively, and only one mouse (1/10) died in the group treated with β-aminopropionitrile combined with 1000 ng/kg/min AngII for 4 weeks. In 6-week-old male ApoE^−/−^ mice that received with 1 g/L BAPN in the drinking water for 3 weeks along with 2500 ng/kg/min AngII infusion via osmotic mini pumps for 2 weeks, the highest model formation rate and relative lower cumulative mortality were noted.

## Introduction

Aortic dissection (AD) is an intractable cardiovascular disease^1,2^, and the diagnosis and treatment of AD remain challenging^3,4^, and to date, other than controlling blood pressure and heart rate, no medications have proven to be effective in halting disease progression, let alone preventing it^5^. Thus, identifying the underlying pathophysiological mechanism of AD becomes the key to innovative diagnostic methods and therapy development. The prerequisite to achieving such goals is the establishment of a cost-effective and suitable animal model. Over the past decades, many animal models of abdominal aortic aneurysm (AAA) have been thoroughly reported; however, AD has received little attention in the scientific community, despite the fact that it is normally thought to derive from aneurysm in the initial phase of AAA animal models (3–7 days), and has thus mostly been treated as a “byproduct” of AAA animal models^6^. Nevertheless, given that the aortic dissection is initiated by an intimal tear, causing a false lumen and rapid expansion in the aorta^2^, it is a more acute and urgent process. This differs from the mechanism of aneurysm formation, which is characterized by aortic dilation that exceeds the normal aortic diameter by more than 50%^7^. Due to accumulating evidence showing the distinct pathophysiological mechanism between AAA and AAD, some researchers began to consider using a higher dose of AngII (2500 ng/kg/min) to create a model of AD, apart from AAA^8^. On the other hand, collagen and elastic fibers are the major components of the media^9^ and their structural destruction can lead to the development and progression of AD. Because beta-aminopropionitrile (BAPN) can inhibit LOX, a key enzyme in the maturation of collagen and elastic fibers^10^, it was thought to be useful in an AD mouse model^11^. Initially, the researchers added BAPN to the drinking water of the mice^12^, and then, some researchers attempted to create advanced models by combining an Ang II pump and a BAPN pump^13^. Asides from that, some new studies used mice challenged with a high-fat diet^14^ or high-salt drinking water^15^ and continuous AngII infusion to induce AD model. The model formation rate of the aortic dissection is not stable in different experiments because the background of the mice used in the modeling process, whether or not they were given the BAPN, and the concentration and duration of AngII were not uniform in each study. Despite recent efforts, a standard, efficient, and cost-effective animal model for AAD is still lacking. Therefore, the purpose of our research is to develop the most appropriate and stable modeling method, by reviewing previous animal experiments and conducting a meta-analysis, which we will combine with our own animal experiments.

## Methods

### systematic review and meta-analysis

#### Data sources and search strategy

A meta-analysis was performed in accordance with the standards set forth by the Preferred Reporting Items for Systematic Reviews and Meta-Analyses (PRISMA) statement. The PubMed were searched using the Mesh “Aneurysm, Dissecting,” “Angiotensin II” and their Entry Terms. Previous meta-analysis and other reviews related to the topic were reviewed to identify studies not included in this search strategy.

#### Inclusion criteria and exclusion criteria

Studies meeting the following criteria were included in the meta-analysis: (1) studies used mice as an animal model. (2) studies used angiotensin II to induce the model of aortic dissection, (3) saline pumps were used as a control in the studies, (4) the incidence of the aortic dissection was reported in each group.

Exclusion criteria included studies that: (1) were reviews or meta-analyses, (2) did not use angiotensin II to induce aortic dissection or did not use a saline pump as control, (3) the studies only mentioned the degree of aortic dilation rather than the success rate of induction of aortic dissection, (4) did not use mice as an animal model, (5) did not have access to full text for quality assessment or data extraction.

#### Data extraction and quality assessment

Data were extracted in duplicate by two independent reviewers (HCJ and WJL), and any disagreements were resolved by consensus. Following information was extracted from studies included: name of the author, year of publication, the number of mice in each group, the number and location of aortic dissections, and the number of aortic dissections ruptured.

#### Data synthesis and statistical analysis

Review manager 5.4 was applied to conduct all data synthesis and statistical analysis. The measurement data were pooled across studies and analyzed using a random-effects meta-analysis model with inverse variance weighting. These are presented as odds ratios (ORs) with 95% confidence intervals (CIs). The magnitude of heterogeneity present was estimated using I^2^ statistics. All P values are 2 tailed with the statistical significance set at 0.05.

### Animal Experiment

#### AD mouse model

**Figure 1:**
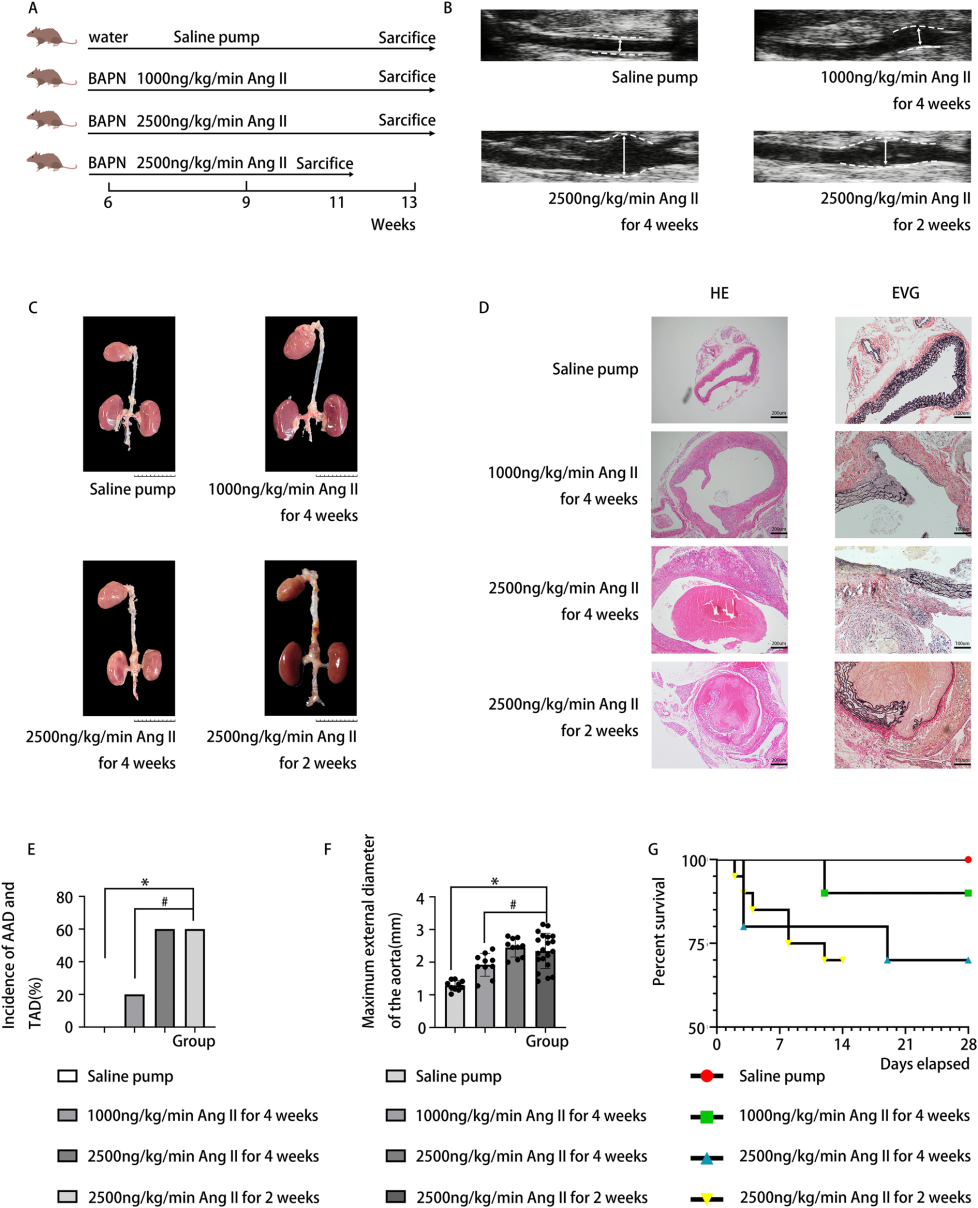
(**A**) Steps of inducing aortic dissection in each group (**B**) Representative ultrasonic image of abdominal aorta of mice in each group (**C**) Representative images of aortas isolated from ApoE−/− mice of each group (**D**) HE staining and EVG staining of abdominal aorta of each group (**E**) Mice treated with BAPN combined with 2500 ng/kg/min Ang II for 2 weeks could significantly increase the incidence of AD compared with saline pump group or treated with 1000 ng/kg/min AngII group. Mice treated with 2500 ng/kg/min Ang II for 4 weeks or 2 weeks had no significant impact on the incidence of AD. (*p < 0.05 vs. saline pump; ^#^p < 0.05 vs. 1000 ng/kg/min AngII, Chi square-test) (**F**) The maximum external diameter of mice treated with BAPN combined with 2500 ng/kg/min Ang II for 2 weeks was significantly increased compared with that in saline pump group or treated with 1000 ng/kg/min AngII group, and the maximum external diameter of the aorta had no significant difference when treated with 2500 ng/kg/min Ang II for 4 weeks or 2 weeks. (*p < 0.05 vs. saline pump; ^#^p < 0.05vs.1000 ng/kg/min AngII, ANOVA-test) (**G**) ApoE−/− Mice were divided into four groups as described earlier. The survival rate was analyzed by Kaplan–Meier survival analysis and compared by the vlog-rank test.

The animal experiments comply with the ARRIVE guidelines (PLoS Bio 8(6), e1000412, 2010) and were approved by the Institutional Animal Research Committee of Tongji Medical College. Our animal experiments were performed in accordance with relevant guidelines and regulations. 6-week-old ApoE^−/−^ male mice (C56BL/6J background) weighing about 20 g (18–22 g) were purchased from Beijing Vital Laboratory Animal Technology Co., Ltd. ApoE^−/−^ mice (C56BL/6J background) were housed at the animal care facility of Tongji Medical College under specific pathogen-free conditions. Male mice (n = 50) were used in our experiment, because the aortic dissection predominantly affects men^15^. These mice were ApoE^−/−^ background and aged 6 weeks. We divided the mice into 4 groups. Mice in the control group (n = 10) were treated with normal drinking water without BAPN combined with saline pump for 4 weeks. Other mice were administered with BAPN dissolved in drinking water (1 g/L) for 3 weeks. The mice were then randomly divided into three groups, with the first group (n = 10) receiving a continuous AngII infusion (1000 ng/kg/min; Sigma-Aldrich, St.Louis, Mo) via osmotic mini pumps (Alzet, Cupertino, CA) for 4 weeks, the second group (n = 10) receiving a continuous AngII infusion (2500 ng/kg/min) for 4 weeks and the third group (n = 20) receiving a continuous AngII infusion (2500 ng/kg/min) for 2 weeks (Fig. 1A). Mice that reached the endpoint of our study were examined by B-ultrasound on the day of tissue harvest to observe the extent of aortic dilation in mice. Additionally, we carried out animal anaesthesia and euthanasia in accordance with American Veterinary Medical Association (AVMA) Guidelines. Specifically, the mice were deeply anesthetized using barbiturates (the euthanasia dose is 3 times the anesthetic dose). After obtaining the mouse aortic tissue, we put the aorta on black paper and put a scale under the aorta, and then we photographed it. Pictures obtained were analyzed with ImageJ software to measure the maximum aortic diameter. The details are as follows: In the software, we first measured the length of the scale (1 cm) and set as the standard length, the diameter of the aortic tissue was then measured in the same image according to the length of the scale. In our study, the locations of thoracic aortic dissection included the aorta above the diaphragm (ascending aorta, aortic arch, descending thoracic aorta), and abdominal aortic dissection was located below the diaphragm.

#### The placement of the pump

The micro osmotic pump was prepared in normal saline the day before and treated overnight at 37 °C. The pump needed to be ready before we could begin the procedure. This included removed the pump from the normal saline and using a syringe to suck up the liquid in it, the AngII solution was prepared according to the dosage (volume of each pump is about 100 µl), putting the pump up, and adding the solution to the pump with a syringe, and install the flow moderator on the pump. And then, the mice were given an intraperitoneal injection of 2% pentobarbital with a dosage of 150 µl, and fix the mouse in a prone position, scissor an incision of about 2 cm at the lower part of the neck near the left side, and blunt separation of the skin and fascia. Next, we placed the assembled micro-osmotic pump under the skin of the mice, sutured the skin, and put each mouse in a separate cage.

#### Tissue acquisition and staining

At the end of the study, the mice were sacrificed with ether anesthesia. The sacrificed mice were perfused with ice-cold PBS to flush as much blood as possible out of the aorta. The aorta was then completely dissected to observe its morphology and measure the external degree of aortic dilatation. Tissues were further fixed in general-purpose tissue fixation solution(Servicebio) for 2 days at 4  °C, embedded in paraffin and processed for sectioning, 4 μm cross-sections were then obtained. Aortic morphology was evaluated using hematoxylin and eosin-stained and van Gieson-stained histological sections (Servicebio Hubei, China). Images were captured using a LEICA DM4000B Microscope (LEICA, 36 Beijing, China). The magnification of the objective lens for HE stained pictures is 10* and the length of the scale bar is 200 μm; the magnification of the objective lens for EVG stained pictures is 20* and the length of the scale bar is 100 μm.

## Results

### meta-analysis

#### Study characteristics

A total of 22 studies enrolling 701 mice were included in this study^8,11–14,16–30^. 449 mice were treated with an AngII pump, and 252 mice were treated with a saline pump. The basic information of each study was shown in Table 1. 190 mice were administered with BAPN dissolved in drinking water or treated with a BAPN pump, and 511 mice were treated without BAPN during the modeling process. In terms of dosage, 174 of them were challenged with 1000 ng/kg/min AngII in the BAPN group, and 153 in the vehicle group, 16 with more than 2000 ng/kg/min AngII in the BAPN group, and 234 in the vehicle group. In terms of duration, 99 of them use AngII for less than 14 days in the BAPN group, and 166 in the vehicle group, 91 of them use AngII for more than 28 days in the BAPN group, and 345 in the vehicle group.

**Table 1:** Basic information of each study.

Author	Year	Age of animals	Strain	Dose of AngII (mg/kg/day)	Duration of treatment (day)	Inclusion of BAPN
Bridge	2017	12–14 weeks	ApoE−/−	1.08	28	No
Colman	2020	Not mentioned	C57BL/6J	1	28	No
Gao	2016	8–9 months old	C57BL/6J	3.6	7	No
Hatipoglu	2020	8 weeks old	C57BL/6J	1.44	7	Yes
Huang	2015	12 weeks old	ApoE−/−	1.44	28	No
Hwang	2014	6 months old	ApoE−/−	1.44	28	No
kanematsu	2010	9 weeks old	C57BL/6J	1.44	42	Yes
Kondo	2020	6–8 weeks old	C57BL/6J	1.44	42	Yes
Kurihala	2012	3 weeks old	FVB	1.44	1	Yes
Liu	2012	12 weeks old	ApoE−/−	1.44	28	No
Liu2	2016	8 months old	ApoE−/−	3.6	7	No
Liu3	2017	12–16 weeks old	C57BL/6J	1.44	28	No
Liu4(1)	2019	8 weeks old	ApoE−/−	1.44	28	No
Liu4(2)	2019	8 weeks old	ApoE−/−	3.6	14	No
Liu4(3)	2019	8 weeks old	ApoE−/−	3.6	14	Yes
Martorell	2016	8 weeks old	ApoE−/−	0.72	28	No
Nogi	2018	6–8 weeks old	ApoE−/−	1.44	28	No
Phillips	2018	9 weeks old	ApoE−/−	1.44	10	No
Qi	2020	10–15 weeks old	C57BL/6J	1.44	28	Yes
Tieu	2009	7–12 months old	C57BL/6J	3.6	7	No
Vorkapic	2015	Not mentioned	ApoE−/−	1.44	28	No
Xanthoulea	2009	10–13 weeks	LDLR−/−	1.44	28	No
Zhou	2020	12 weeks old	C57BL/6J	3	28	No
Zou	2020	8 weeks old	C57BL/6J	2.88	28	No

#### BAPN could increase the success rate of the mouse model of AAD

Our result of the meta-analysis showed that AngII can successfully induce the formation of the aortic dissection in mice (OR 20.83, 95% CI 10.98–39.51, P < 0.00001, I2 = 0%) (Table 2). Meanwhile, when compared to mice treated without BAPN during the modeling process, the BAPN-treated mice had a higher success rate for the mouse AD model (OR 49.34, 95% CI 14.04–173.34, P 0.00001, I2 = 0%) than the mice treated without BAPN (OR 15.23, 95% CI 7.20–32.21, P 0.00001, I^2^ = 0%) (Table 3).

**Table 2:**
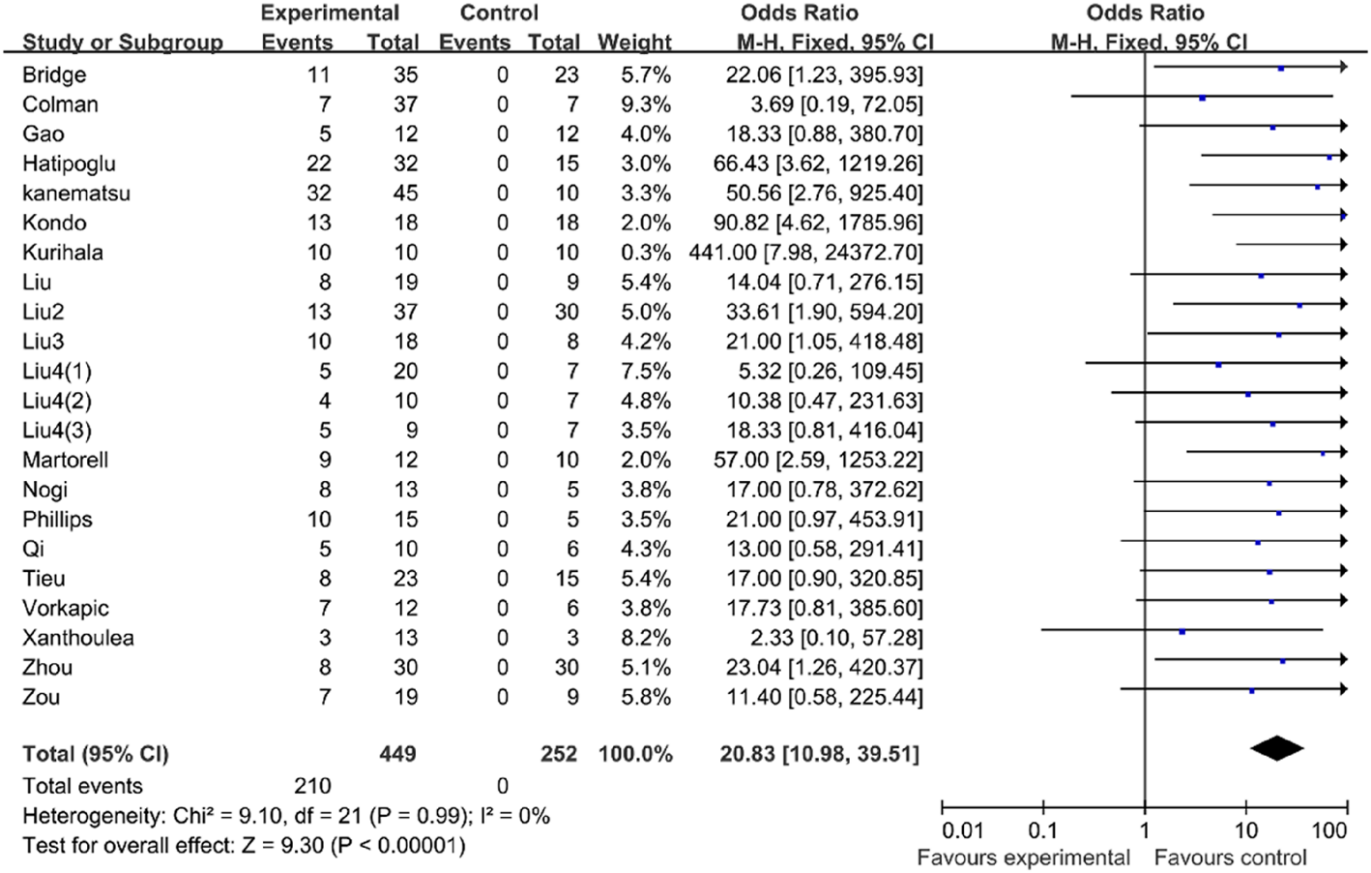
AngII can successfully induce the formation of the aortic dissection in mice.

**Table 3:**
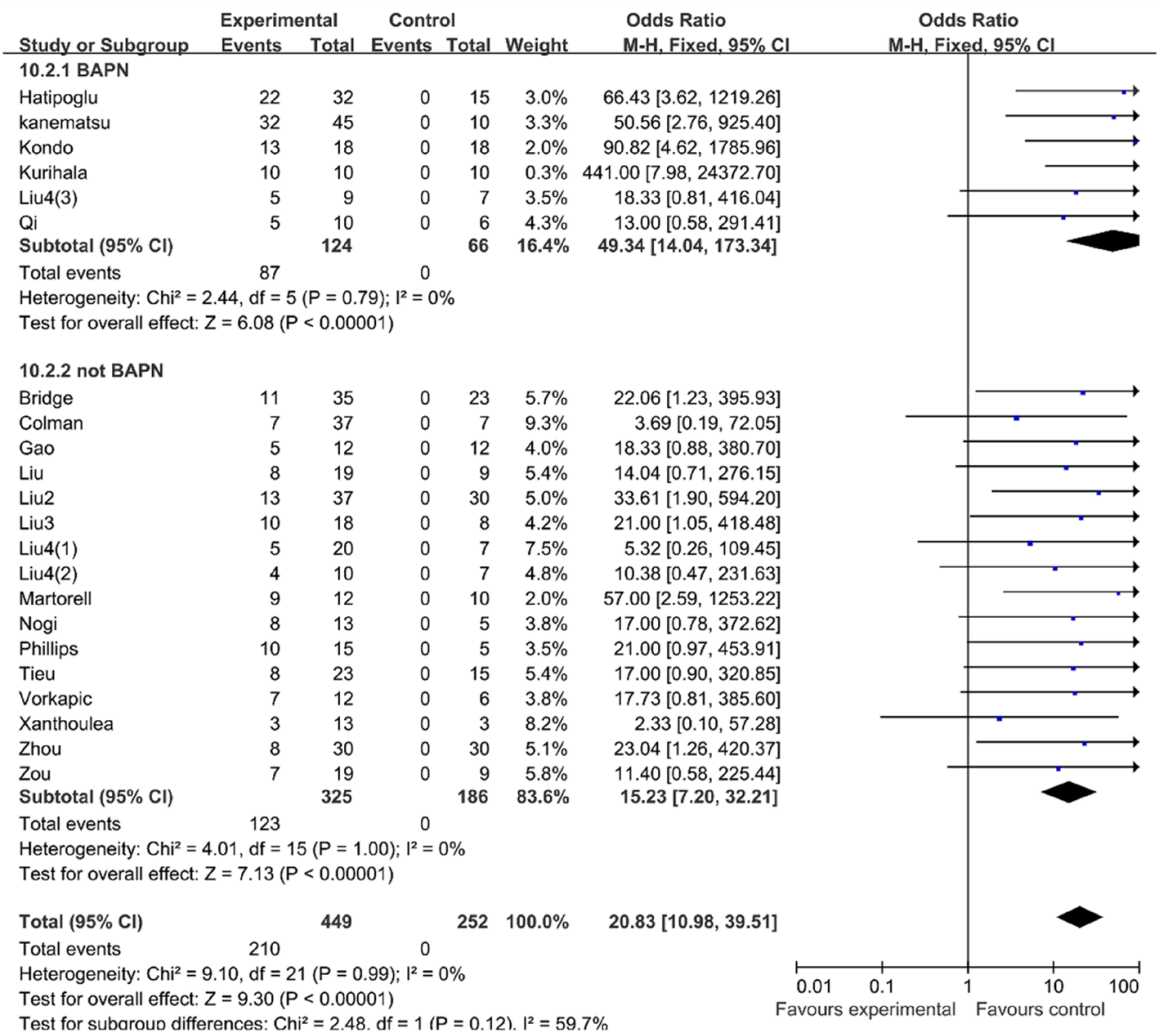
BAPN can increase the success rate of AD model in mice.

#### Higher dose AngII could increase the success rate of the mouse AD model

In consideration of the previous result that BAPN can influence the success rate of the AD model, we divided the research into two groups, namely mice challenged with BAPN (BAPN group) and no BAPN was used during the research (vehicle group). In the vehicle group, the mice challenged with more than 2000 ng/kg/min AngII (OR 18.80, 95% CI 5.62–62.87, P < 0.00001, I^2^ = 0%) could increase the success rate of the mouse AD model compared with the mice challenged with 1000 ng/kg/min AngII (OR 11.81, 95% CI 3.75–37.20, P < 0.0001, I^2^ = 0%) (Table 4). However, in the BAPN group, the success rate in the mice challenged with more than 2000 ng/kg/min AngII (OR 18.33, 95% CI 0.81–416.04, P = 0.07) decreased compared with the mice challenged with 1000 ng/kg/min AngII (OR 57.73, 95% CI 14.37–231.97, P < 0.0001, I^2^ = 0%) (Table 5).

**Table 4:**
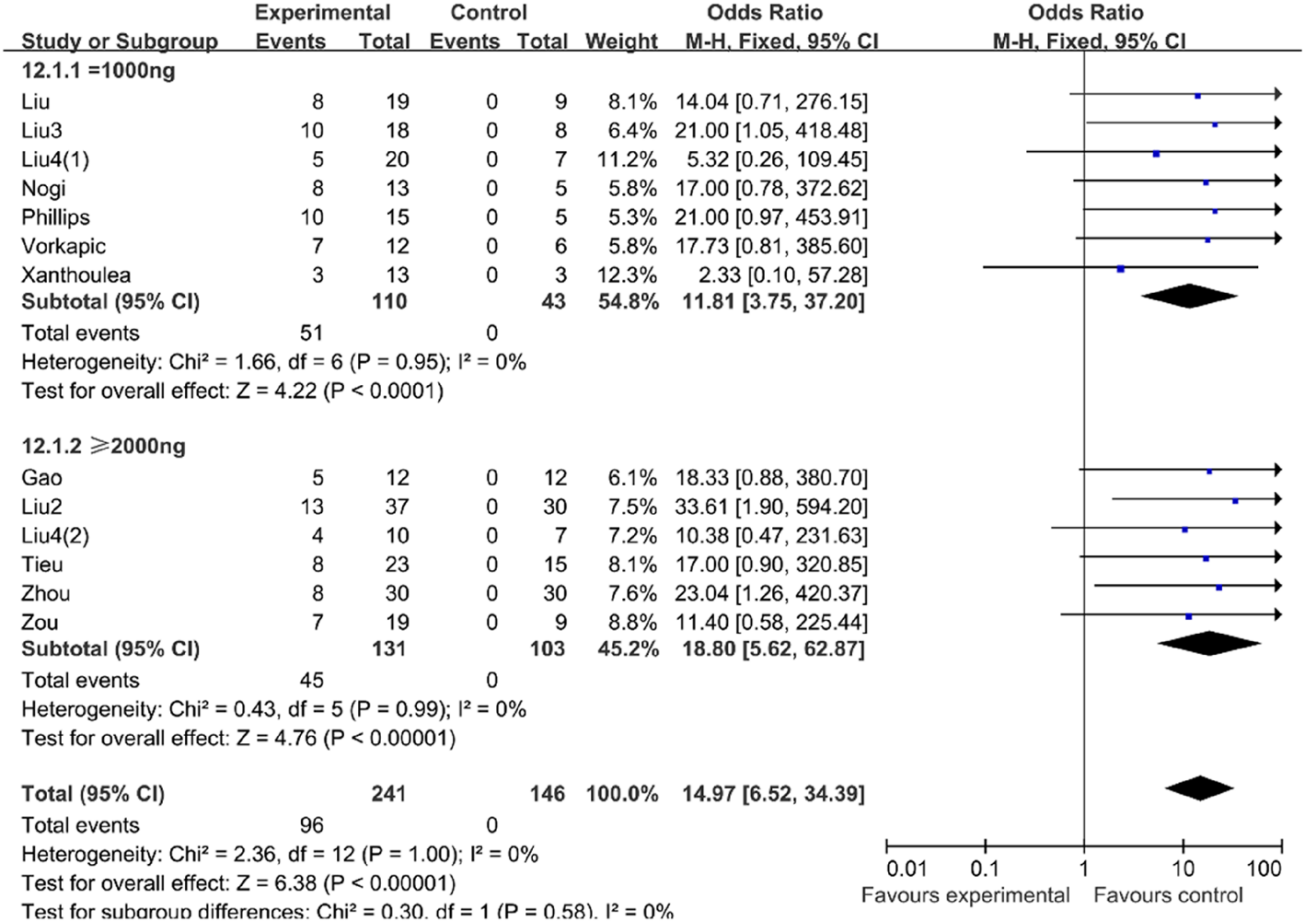
In the vehicle group, the mice challenged with more than 2000 ng/kg/min AngII could increase the success rate of the AD model compared with the mice challenged with 1000 ng/kg/min AngII.

**Table 5:**
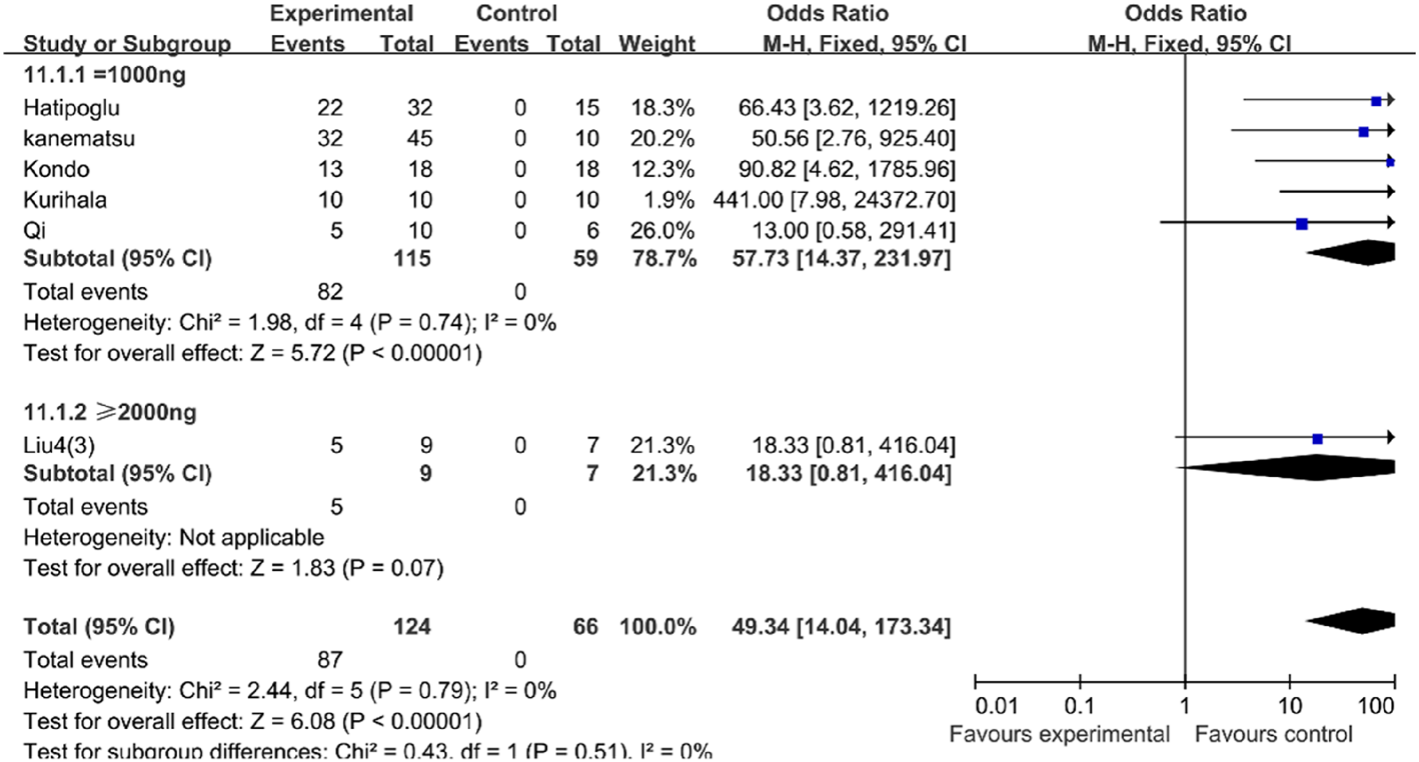
In the BAPN group, the success rate in the mice challenged with more than 2000 ng/kg/min AngII decreased compared with the mice challenged with 1000 ng/kg/min AngII.

#### Prolonged administration of AngII could not significantly improve the success rate of the mouse AD model

Through the meta-analysis, we found that compared with the mice challenged with AngII less than 14 days (OR 41.40, 95% CI 8.65–198.07, P < 0.0001, I^2^ = 0%), the mice challenged with AngII longer than 28 days (OR 65.83, 95% CI 8.10–535.14, P < 0.0001, I^2^ = 0%) could increase the success rate in the BAPN group (Table 6). However, the result in the vehicle group was reversed. The success rate of the mice challenged with AngII longer than 28 days (OR 13.42, 95% CI 5.45–33.03, P < 0.0001, I^2^ = 0%) decreased compared with the mice challenged with AngII less than 14 days (OR 20.09, 95% CI 5.25–76.91, P < 0.0001, I^2^ = 0%) (Table 7).

**Table 6:**
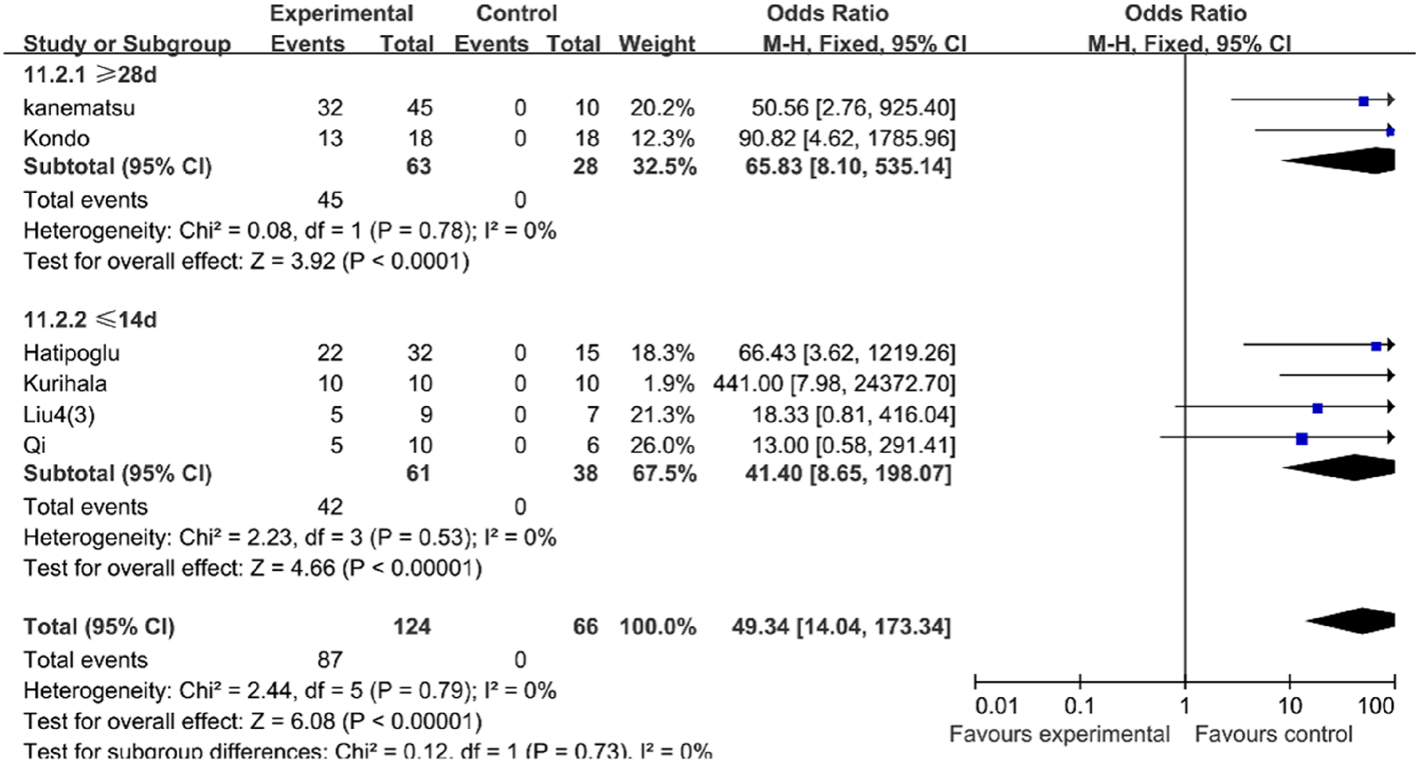
In the BAPN group, the mice challenged with AngII longer than 28 days could increase the success rate of AD model compared with the mice challenged with AngII less than 14 days.

**Table 7:**
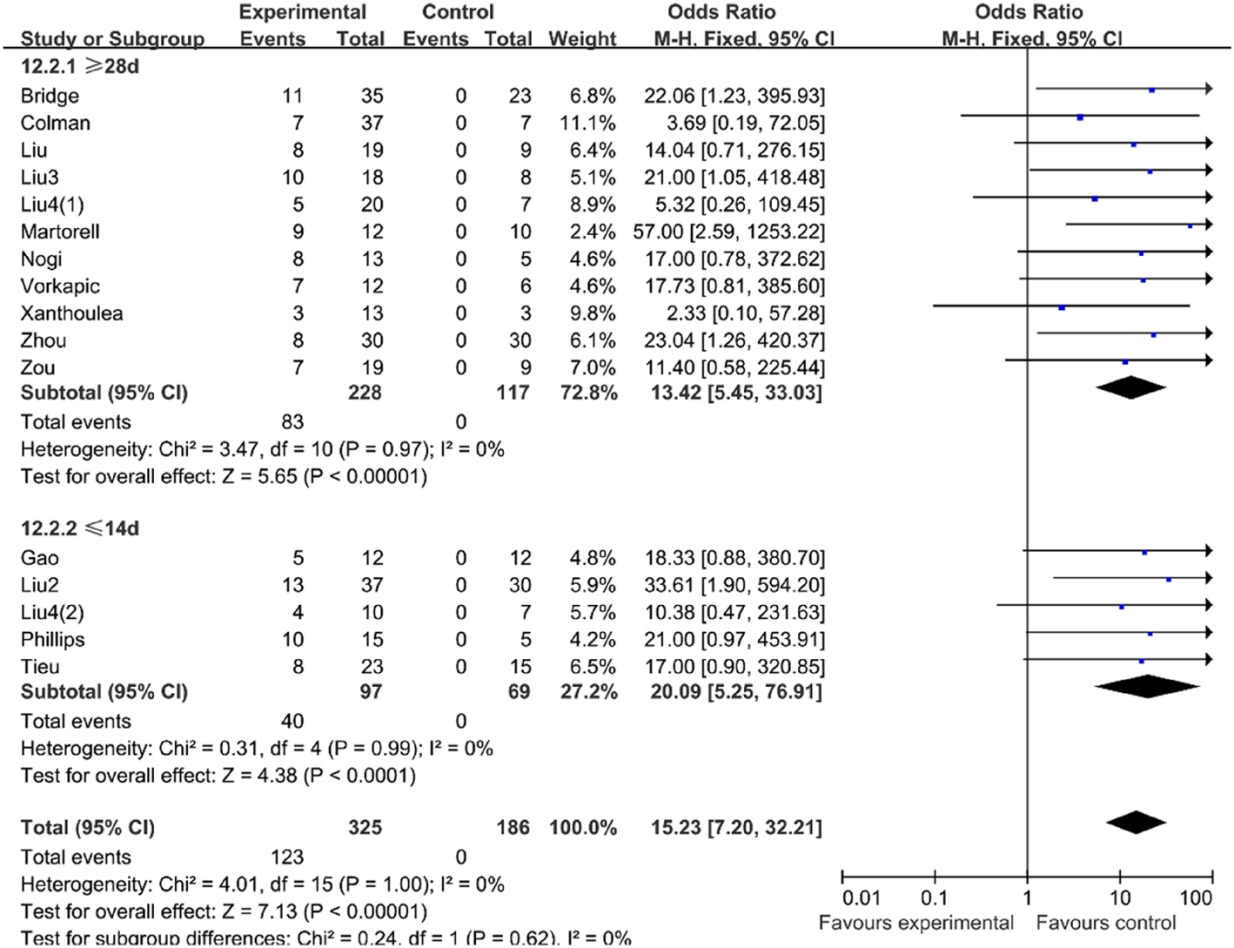
In the vehicle group, the success rate of the mice challenged with AngII longer than 28 days decreased compared with the mice challenged with AngII less than 14 days.

### Animal experiment

To determine the presence of aortic dissection, we firstly performed B-ultrasonography before dissecting the mice to observe the extent of aortic dilation of their abdominal aorta and thoracic aorta (Fig. 1B and Supplementary Fig. 1A). Then through the gross observation of the aortic tissue dissected from mouse anatomy and HE and EVG staining(Fig. 1C,D and Supplementary Fig. 1D), we found that the incidence of AD in the group infused with AngII at a dosage of 2500 ng/kg/min for 4 weeks was 60% (6/10, 4 thoracic aortic dissection (TAD) and 5 abdominal aortic dissection (AAD) ) and was also 60% (12/20, 7 TAD and 11 AAD) in the group infused with AngII at a dosage of 2500 ng/kg/min for 2 weeks, while the group infused with 1000 ng/kg/min AngII for 4 weeks was 20% (2/10, 1 TAD and 2 AAD) (Fig. 1E and Supplementary Fig. 1B,C). The measurement of the isolated aorta revealed that the maximum external diameter of the aorta in the group infused with 2500 ng/kg/min AngII for 2 weeks (2.346 ± 0.539 mm) was significantly increased compared with that in the group infused with 1000 ng/kg/min AngII for 4 weeks (1.921 ± 0.352 mm) and group infused with saline for 4 weeks (1.285 ± 0.152 mm), however, the maximum external diameter of the aorta in the group infused with 2500 ng/kg/min AngII for 2 weeks had no significant increase compared to the group infused with 2500 ng/kg/min AngII for 4 weeks(2.447 ± 0.287 mm) (Fig. 1F). In the group treated with BAPN combined with 2500 ng/kg/min AngII for 4 weeks, two mice died three days after the pump was implanted and one died 19 days after the implantation. In contrast, in the group treated with BAPN combined with 2500 ng/kg/min AngII for 2 weeks, there were one mouse that died 2 days, 3 days, 4 days and 12 days after the pump was implanted respectively and two mice died 8 days after the implantation. Meanwhile, one mouse died 12 days after the pump was implanted in the group treated with BAPN combined with 1000 ng/kg/min AngII for 4 weeks (Fig. 1G).

## Discussion

By reviewing previous studies, conducting a meta-analysis and animal experiments, we came to the conclusion that mice fed with 1 g/L BAPN in drinking water for 3 weeks combined with an infusion of 2500 ng/kg/min AngII via osmotic-mini pumps for 2 weeks has the highest model formation rate and lower cumulative mortality.

Due to the inconsistency of previous modeling methods for aortic dissection and the lack of a stable modeling rate, we reviewed previous studies involving the modeling of aortic dissection and conducted a meta-analysis. The result of our meta-analysis showed that BAPN could increase the success rate of the mouse AAD model, consequently, in the following meta-analysis, we divided the existing research into two groups, the BAPN group, and the vehicle group. When we focused on the dosage of the AngII, as we expected, we found that mice administrated with more than 2000 ng/kg/min AngII in the vehicle group had a higher success rate of modeling. However, the BAPN group had a contrary result, which may be due to the fact that the number of studies in this group was too limited to draw a credible conclusion. In terms of the use time of the AngII, we discovered that in the BAPN group the success rate of modeling would increase with the prolonged administration of AngII, while the result of the vehicle group was the opposite. Therefore, considering that longer use time of AngII may not guarantee a higher success rate of modeling and may lead to increased cumulative mortality, we considered 14 days as our modeling time.

To verify the results of the meta-analysis and to find a more reliable modeling method, we established the AD mouse model by combining the BAPN and 1000 ng/kg/min or 2500 ng/kg/min AngII pump. After evaluating our mouse model, we were able to show that the group that was administered AngII at a dose of 2500 ng/kg/min for 2 weeks had a significantly higher incidence of AD formation compared to mice treated with BAPN combined with 1000 ng/kg/min AngII for 4 weeks and a similar cumulative survival rate compared to mice treated with BAPN combined with 2500 ng/kg/min AngII for 4 weeks. Hence, we established a reliable and easy-to-use mouse model for investigating the pathological mechanisms of AD.

Aortic dissection and rupture are mainly characterized by extensive vascular inflammation and disruption of the structural extracellular matrix, among which pro-inflammatory and pro-apoptotic responses are the central biological process in thepathogenesis of AD. Besides, oxidative stress also plays a crucial role in the pathogenesis of AD^31^, and some studies have shown that vascular smooth muscle cell (VSMC) apoptosis could be induced by oxidative stress and then increased the risk of dissection^32^. In addition, the significance of endothelial dysfunction during the onset of aortic dissection has been addressed. Ishizawa et al. demonstrated that L-NAME-induced endothelial dysfunction may trigger AD in mice^33^. Although the biological mechanisms that cause aortic dissection are diverse and interrelated, AngII is involved in most of these pathological processes. Therefore, most of the models chose AngII as the induction drug. However, different studies have selected mice of different weeks of age and backgrounds, and the doses and duration of AngII are also multitudinous. In our earlier study^21^ we found that the development of AD (suprarenal more often than thoracic) was more often found in older mice (7- to 12-month-old) after angiotensin II infusion. Besides, there were also a variety of AngII dose choices in the AD model. By reviewing previous studies, we found that the most commonly used dose was 1000 ng/kg/min and greater than 2000 ng/kg/min. To more accurately compare the effects of different doses of AngII and their use time on the success rate of inducing mouse AD model, we carried out the meta-analysis and concluded that using AngII less than 14 days at a dose of greater than 2000 ng/kg/min could induce a higher incidence of AD formation and a lower cumulative survival rate, and we validated this result with our own animal experiments.

However, AngII alone resulted in a low incidence of AD. Kurihara T first showed that when combined with higher doses of BAPN with longer durations, AD would occur in 100% of the mice within 24 h of the start of AngII loading, whereas in mice treated with BAPN combined with AngII, neutrophils infiltrate the aortic intima, invariably triggering AD via metalloproteinase-9 production. Since then, a large number of studies have also confirmed the effect of BAPN in the AD model. BAPN treatment triggers TAD formation mainly through medial degeneration accompanied by VSMC apoptosis and vascular inflammation, which is consistent with the characteristics of human TAD. Therefore, BAPN treatment is the most accepted animal model of TAD. The administration of AngII and BAPN to mice pharmacologically induces acute aortic aneurysm or dissection by causing hypertension and medial degradation. More and more experiments are focusing on aortic dissection induced by BAPN; however, the application methods of BAPN vary, some experiments add BAPN to drinking water, while others use a BAPN pump. Additionally, the dosage and use time of BAPN are also different. In our meta-analysis, we only focused on whether BAPN was used or not. According to the results of our meta-analysis, the incidence of AD was greatly increased by BAPN, which is also consistent with previous studies. Therefore, we determined the scheme of co-induction of AngII and BAPN.

There are also great differences in the selection of modeling time. Most schemes focus on 7 days, 14 days, and more than 28 days. Because the appearance of an aortic wall tear, as detected by the Evans blue perfusion, occurred on day 4 of AngII infusion. Meta-analysis shows that there was no statistically significant difference in incidence between the 14-day model and the 28-day model. However, the longer modeling time results in a greater cost and increases the mortality of the model mice. Therefore, we chose 14 days as our modeling time. Reducing the use time of AngII from 28 to 14 days is not only for cost savings but also because we have verified that shortening the modeling time can achieve similar modeling success rates, while the cumulative mortality of mice decreases, which may allow researchers to obtain more aortic tissue from mice with aortic dissection, which is necessary for the study of aortic dissection.

Nonetheless, our article has some limitations. Firstly, despite our efforts to screen, we could not guarantee that each of the included articles of our meta-analysis was consistent in their assessment of the aortic dissection of the mice. Secondly, the modeling method we recommend is solely based on the existing literature, and as a result, it has a higher modeling success rate, and lowers cumulative mortality.

To sum up, our study found a more stable modeling method for aortic dissection with lower cumulative mortality, which provides a basis for future research on aortic dissection.

## Supplementary Information

Supplementary Figure 1.

## Data Availability

The datasets used and/or analyzed during the current are study available from the corresponding author upon reasonable request.
