# Supplementary figures and images for: Establishment of a meta-analysis based novel aortic dissection mouse model
Source: Sci Rep. 2022 Dec 12;12:21434. doi: 10.1038/s41598-022-25369-x (PMC9744727; doi:10.1038/s41598-022-25369-x)

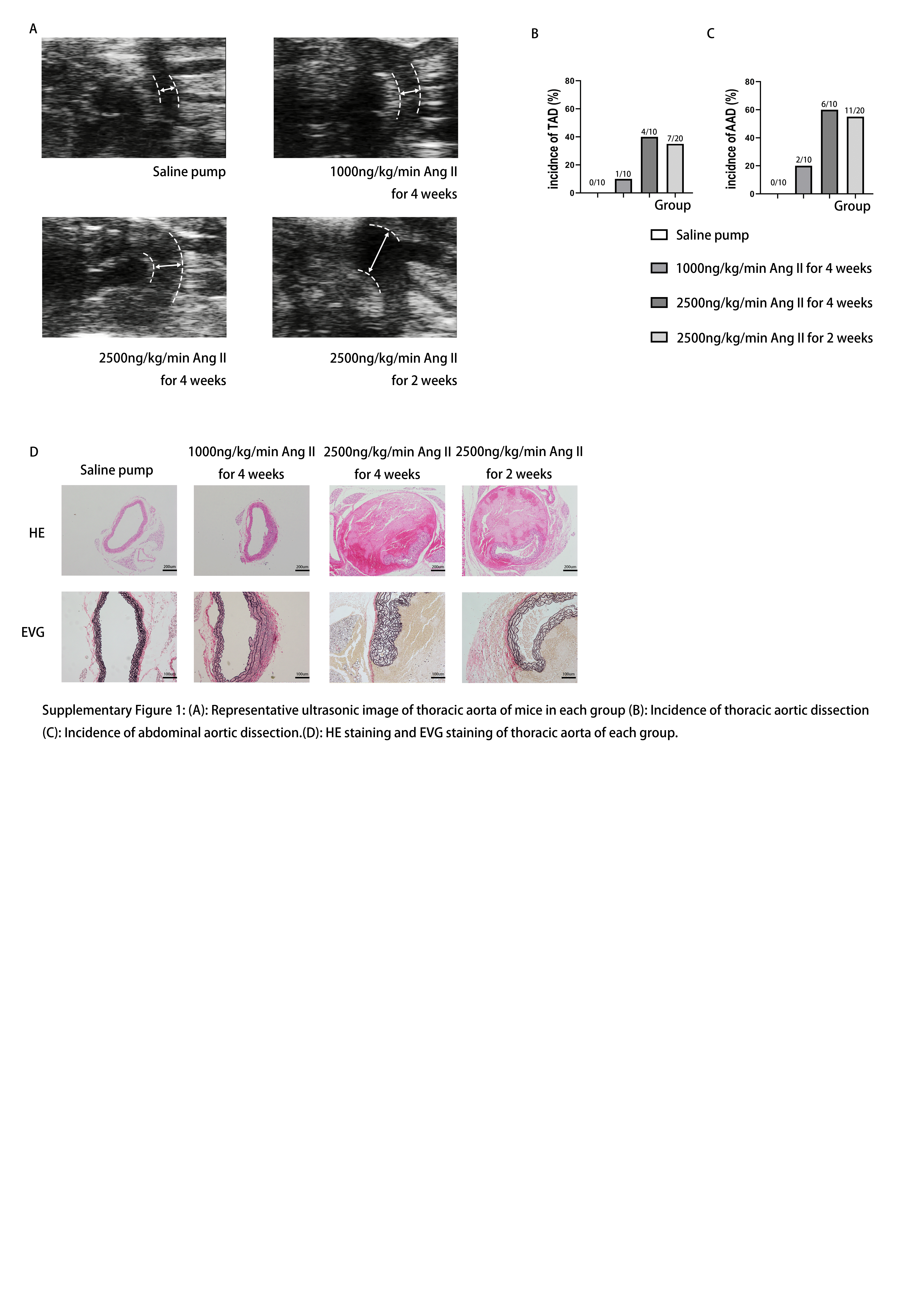

Supplement: Supplementary file 1 — Supplementary Figure 1. [file 41598_2022_25369_MOESM1_ESM.png]
